# Coexisting Moderate-to-Severe Tricuspid Regurgitation and Renal Dysfunction Identify a High-Risk Group After Transcatheter Aortic Valve Replacement

**DOI:** 10.3390/jcdd13100520

**Published:** 2026-10-16

**Authors:** Philipp Lauten, Birgit Markus, Georgios Chatzis, Styliani Syntila, Bernhard Schieffer, Klevis Mihali, Julian Kreutz

**Affiliations:** 1 Department of Cardiology, Angiology, and Intensive Care Medicine, Philipps-Universität Marburg, Baldingerstraße, 35043 Marburg, Germany; 2 Department of Cardiology, Heart Center, Central Hospital of Bad Berka, 99437 Bad Berka, Germany

**Keywords:** transcatheter aortic valve replacement, tricuspid regurgitation, renal dysfunction, cardiorenal phenotype, risk stratification

## Abstract

Background: Moderate-to-severe tricuspid regurgitation (TR) and renal dysfunction are each associated with adverse outcomes after transcatheter aortic valve replacement (TAVR). We investigated whether their coexistence identifies a clinically relevant high-risk group. Methods: In this retrospective two-centre cohort, patients undergoing TAVR were classified into four groups according to moderate-to-severe TR and preprocedural estimated glomerular filtration rate < 45 mL/min/1.73 m^2^. The primary outcome was one-year all-cause mortality. Multivariable logistic regression adjusted for prespecified clinical covariates, and multiplicative interaction between TR and renal dysfunction was assessed. Results: Among 2227 patients, 1456 had neither condition, 324 isolated renal dysfunction, 315 isolated TR, and 132 both conditions. Patients with the combined phenotype had the highest rates of in-hospital mortality, 30-day mortality, acute kidney injury, and renal replacement therapy (all *p* < 0.001). One-year mortality increased progressively across groups (6.7%, 10.6%, 11.3%, and 22.9%; *p* < 0.001). After adjustment (*n* = 1704), the combined phenotype was associated with higher one-year mortality compared with neither condition (OR 2.91, 95% CI 1.62–5.23; *p* < 0.001), isolated TR (OR 2.17, 95% CI 1.16–4.07; *p* = 0.015), and isolated renal dysfunction (OR 2.32, 95% CI 1.16–4.61; *p* = 0.017). No significant multiplicative interaction was observed (*p* = 0.187). Conclusions: Coexisting moderate-to-severe TR and renal dysfunction identify a clinically relevant high-risk group after TAVR, with higher adjusted one-year mortality than either condition alone. Combined assessment of TR and renal function may improve preprocedural risk stratification.

## 1. Introduction

Transcatheter aortic valve replacement (TAVR) has become an established treatment for severe aortic stenosis across an increasingly broad spectrum of age and surgical risk. The 2025 European guidelines further extended TAVR to anatomically suitable patients aged ≥70 years with tricuspid aortic valve anatomy, irrespective of their estimated surgical risk [1]. As procedural safety and indications have expanded, attention has shifted towards identifying patients who remain at risk despite correction of the valvular obstruction. This is particularly relevant in the elderly, multimorbid TAVR population, in whom functional status, concomitant cardiac disease, and extracardiac organ dysfunction influence prognosis. Previous studies have consistently identified advanced NYHA functional class, atrial fibrillation, peripheral artery disease, and renal dysfunction as important predictors of adverse outcome [2,3]. Moderate-to-severe tricuspid regurgitation (TR) represents an important marker of advanced cardiac damage in patients with aortic stenosis. Chronic left-sided pressure overload, pulmonary hypertension, right ventricular remodelling, and AF may promote tricuspid annular dilatation and secondary TR. In a contemporary registry including more than 300,000 patients undergoing TAVR, increasing TR severity was associated with progressively higher mortality and poorer health status at one year [4]. Similarly, severe baseline TR and persistent severe TR after TAVR were associated with increased mortality during follow-up [5]. Although TR may improve following relief of the left-sided valvular obstruction, its course remains variable, and persistent right-sided volume overload may limit the clinical benefit achieved through TAVR. Renal dysfunction is likewise common among patients undergoing TAVR and is associated with increasing mortality and bleeding according to its severity [6]. Although renal function remains stable or improves in a substantial proportion of patients after TAVR, advanced preprocedural impairment continues to identify patients with reduced physiological reserve and an unfavourable prognosis [7]. Importantly, the association between TR and renal dysfunction may reflect more than the parallel accumulation of comorbidities. Elevated right atrial and central venous pressures can increase renal venous pressure and reduce the effective renal perfusion gradient. Conversely, impaired renal function promotes sodium and fluid retention, neurohormonal activation, and systemic congestion, potentially aggravating right ventricular loading conditions and TR. This bidirectional interaction has been described as a cardiorenal triangle linking TR, right ventricular dysfunction, and renal congestion [8,9]. Despite this biological interdependence, TR and renal dysfunction have predominantly been evaluated as separate prognostic variables after TAVR. Recent findings in patients with low-flow, low-gradient aortic stenosis suggest that advanced chronic kidney disease and composite right-sided extravalvular damage may jointly identify a particularly vulnerable subgroup [10]. However, that analysis was restricted to low-flow, low-gradient aortic stenosis and defined right-sided damage using a composite of pulmonary hypertension, TR, and right ventricular dysfunction. Whether coexisting TR and renal dysfunction identify a clinically relevant high-risk group across the broader TAVR population remains unclear. We therefore examined the individual and combined associations of moderate-to-severe TR and preprocedural eGFR < 45 mL/min/1.73 m^2^ with one-year mortality and assessed potential multiplicative interaction.

## 2. Materials and Methods

### 2.1. Study Design and Population

This retrospective, observational, two-centre cohort study included consecutive patients who underwent TAVR at the University Hospital of Marburg and the Central Hospital of Bad Berka, Germany, between January 2017 and December 2022. All consecutive TAVR procedures during the study period were eligible. Eligibility was not restricted according to chronic dialysis status, bicuspid aortic valve morphology, valve-in-valve treatment, or procedural urgency, and no additional clinical or procedural exclusion criteria were applied. All patients underwent a comprehensive preprocedural evaluation, including assessment of their medical history, laboratory parameters, electrocardiography, transthoracic echocardiography, coronary status, and computed tomography for procedural planning. The indication for TAVR and the procedural strategy were determined by the multidisciplinary Heart Team at each centre. Periprocedural and postprocedural management followed established institutional standards. For the present analysis, patients were required to have both a preprocedural assessment of TR and an available preprocedural eGFR. Patients missing either variable were excluded from phenotype-based analyses, while those without documented one-year mortality status were excluded from the primary outcome analysis.

### 2.2. Data Collection and Definitions

Baseline data included demographic characteristics, NYHA functional class, cardiovascular risk factors, relevant comorbidities, preprocedural laboratory and echocardiographic findings, computed tomography measurements, procedural characteristics, and in-hospital complications. Renal function was assessed using the preprocedural eGFR, calculated using the Chronic Kidney Disease Epidemiology Collaboration (CKD-EPI) equation [11]. The most recent eGFR value obtained during the index hospitalization before TAVR was used for analysis. Renal dysfunction was defined a priori as an eGFR < 45 mL/min/1.73 m^2^, corresponding to CKD stage G3b or worse and thereby identifying patients with at least moderately to severely reduced kidney function. Patients with an eGFR ≥ 45 mL/min/1.73 m^2^ constituted the reference category. Values reported as >60 mL/min/1.73 m^2^ were set to 60 for continuous analyses without affecting phenotype classification. Preprocedural TR and MR severity were assessed by transthoracic echocardiography and graded according to the European recommendations applicable during the study period [12]. Regurgitation grade ≥ 2, corresponding to moderate or severe regurgitation in the contemporary three-grade classification, was considered at least moderate. Patients were subsequently assigned to four mutually exclusive phenotypes: neither condition, isolated renal dysfunction, isolated moderate-to-severe TR, or coexisting moderate-to-severe TR and renal dysfunction. Procedural contrast volume was not systematically recorded and could therefore not be accounted for when evaluating post-TAVR AKI.

### 2.3. Study Outcomes

The primary outcome was one-year all-cause mortality after TAVR. Mortality status was obtained from institutional medical records and documented follow-up information. One-year vital status was recorded as a fixed binary outcome. Dates of death and reliable dates of last known clinical contact were not systematically captured in the analytical database; therefore, individual follow-up durations and censoring times could not be reconstructed. Secondary outcomes included postprocedural length of hospital stay, in-hospital mortality, 30-day all-cause mortality, acute kidney injury (AKI), and initiation of continuous venovenous hemodiafiltration for AKI.

AKI was identified retrospectively from available serial serum creatinine measurements obtained during hospitalisation and defined according to the creatinine-based KDIGO criteria as an increase in serum creatinine of ≥0.3 mg/dL within 48 h or to ≥1.5 times baseline within 7 days. Postprocedural creatinine measurements were obtained as part of routine clinical care rather than according to a study-specific surveillance protocol. Consequently, the timing and frequency of creatinine assessments were clinically driven and were not systematically standardised or recorded across the four phenotypes. Documented clinical diagnoses were reviewed for verification. Urine output criteria were not applied. Secondary outcomes were analysed on an unadjusted and exploratory basis.

### 2.4. Statistical Analysis

Categorical variables are presented as numbers and percentages. Continuous variables are reported as mean ± standard deviation or median with interquartile range (IQR), depending on their distribution. Comparisons across the four phenotypes were performed using Pearson’s chi-squared test for categorical variables. Continuous variables were compared using the Kruskal–Wallis test. When the overall Kruskal–Wallis test was significant, pairwise comparisons were performed with Bonferroni adjustment for multiple testing. Analyses involving sparse secondary outcome tables were considered exploratory and interpreted cautiously when expected cell frequencies were <5. Because the primary endpoint was defined as fixed one-year vital status and reliable dates of death or last known follow-up were unavailable, one-year mortality was analysed as a binary endpoint rather than using censoring-based time-to-event methods. One-year mortality rates were initially compared across the four phenotypes using Pearson’s chi-squared test. Univariable binary logistic regression was subsequently performed to estimate the unadjusted association between phenotype and one-year mortality. Patients with neither condition constituted the reference group. To directly compare the combined phenotype with each isolated phenotype, the multivariable model was additionally repeated using isolated moderate-to-severe TR and isolated renal dysfunction, respectively, as the reference category.

The association between the TR–renal phenotype and one-year all-cause mortality was evaluated using multivariable binary logistic regression. The model included the four-level TR–renal phenotype, age, sex, NYHA functional class, left ventricular ejection fraction (LVEF) ≤ 45%, AF, chronic obstructive pulmonary disease, PAD, diabetes mellitus, and at least moderate mitral regurgitation. NT-proBNP was not included in the primary multivariable model because it was not part of the prespecified covariate set, is substantially influenced by renal function and haemodynamic congestion, and may therefore partly reflect the phenotype under investigation rather than represent an independent confounder. Its inclusion would additionally have reduced the complete-case sample because of missing measurements. All variables were entered simultaneously, and the analysis was performed using complete cases. Results are reported as odds ratios (OR) with 95% confidence intervals (CI). In additional analyses, moderate-to-severe TR and renal dysfunction were first evaluated separately in univariable logistic regression models. To assess their independent prognostic contributions, a separate multivariable model included moderate-to-severe TR and renal dysfunction simultaneously as binary predictors, while retaining patients with both conditions and adjusting for the same prespecified covariates as the primary phenotype model. To assess whether the association between TR and renal dysfunction exceeded their individual contributions on the multiplicative scale, a separate model included the main effects of moderate-to-severe TR and renal dysfunction together with a TR × renal dysfunction interaction term. This model was adjusted for the same prespecified covariates as the primary model. A statistically significant interaction term was considered evidence of multiplicative effect modification. Model calibration was assessed using the Hosmer–Lemeshow goodness-of-fit test. Overall model significance was evaluated using the omnibus likelihood-ratio test, and explanatory performance was summarised using Nagelkerke’s R^2^. Multicollinearity was assessed using tolerance values and variance inflation factors. As sensitivity analyses, renal function was additionally evaluated as a continuous variable, expressed per 10 mL/min/1.73 m^2^ decrease in eGFR, in univariable and multivariable logistic regression models. The multivariable model included continuous eGFR and moderate-to-severe TR simultaneously and was adjusted for the same prespecified covariates as the primary model. In addition, the primary model was repeated after replacing the dichotomised LVEF variable (≤45% versus >45%) with the original four-level classification of preserved, mildly reduced, moderately reduced, or severely reduced LVEF, and after excluding patients receiving chronic dialysis before TAVR. In the latter analysis, the primary phenotype model and the TR × renal dysfunction interaction model were repeated using the same prespecified covariates. Missing data were not imputed. Descriptive comparisons were conducted using available cases, whereas multivariable analyses were performed using complete-case analysis. The number of observations available for each variable is reported in the corresponding tables. All tests were two-sided, and *p* < 0.05 was considered statistically significant. Secondary and sensitivity analyses were considered exploratory. Statistical analyses were performed using IBM SPSS Statistics, version 29.0.2.0 (IBM Corp., Armonk, NY, USA), and figures were generated using GraphPad Prism, version 11 (GraphPad Software, Boston, MA, USA).

### 2.5. Ethics Approval and Study Registration

The study was approved by the Ethics Committee of Philipps University of Marburg (reference RS 22/65; 27 October 2022) and the Ethics Committee of the Medical Association of Thuringia (13 July 2023).

## 3. Results

### 3.1. Study Population and Phenotype Distribution

Of 2256 consecutive patients undergoing TAVR between January 2017 and December 2022, 29 were excluded from the phenotype-based analysis because of missing preprocedural TR assessment (*n* = 27) or eGFR data (*n* = 2). The resulting phenotype cohort comprised 2227 patients: 1456 (65.4%) had neither condition, 324 (14.5%) had isolated renal dysfunction, 315 (14.1%) had isolated moderate-to-severe TR, and 132 (5.9%) had both conditions. One-year mortality status was available for 1774 patients overall, of whom 1746 had complete phenotype data and constituted the primary outcome cohort. After excluding 42 patients with incomplete covariate data, 1704 patients were included in the multivariable analysis (Figure 1).

### 3.2. Baseline and Procedural Characteristics

Baseline characteristics across the four TR–renal phenotypes are summarized in Table 1. Phenotypes involving renal dysfunction were characterized by a higher prevalence of diabetes mellitus, coronary artery disease and prior myocardial infarction, as well as lower haemoglobin levels and greater estimated procedural risk. Phenotypes involving moderate-to-severe TR more frequently showed AF, pre-existing permanent pacemakers, reduced LVEF, concomitant mitral regurgitation, and signs of congestion. Overall, the greatest disease burden was observed in patients with both conditions, who also had more advanced functional limitation and markedly higher NT-proBNP levels.

The four principal transcatheter valve platforms were the Edwards SAPIEN 3 balloon-expandable system (*n* = 1225), the Medtronic CoreValve/Evolut self-expanding system (*n* = 585), the Boston Scientific SYMETIS ACURATE neo TF self-expanding system (*n* = 386), and the Boston Scientific LOTUS Edge mechanically expanded system (*n* = 28). Valve-platform distribution did not differ significantly across the four phenotypes (*p* = 0.072). Transfemoral access was used in 2201 patients (98.8%), without significant differences between the groups (*p* = 0.942). Catecholamine support for >6 h was required in 57 (3.9%), 37 (11.4%), 8 (2.5%), and 15 (11.4%) patients with neither condition, isolated renal dysfunction, isolated moderate-to-severe TR, and both conditions, respectively (*p* < 0.001). Clinical outcomes are summarized in Table 2.

### 3.3. Primary Outcome

One-year vital status was available for 1746 of the 2227 patients included in the phenotype-based cohort (78.4%), whereas it was unavailable for 481 patients (21.6%). Baseline characteristics according to one-year follow-up availability are presented in Supplementary Table S1. Several baseline characteristics differed between patients with and without available one-year vital status, including age, NYHA functional class, comorbidity burden, renal dysfunction, and echocardiographic characteristics, indicating the potential for selection bias related to incomplete follow-up. Among the 1746 patients with complete phenotype and one-year outcome data, 155 died within one year. One-year mortality differed significantly across the four phenotypes, occurring in 6.7% of patients with neither condition, 10.6% with isolated renal dysfunction, 11.3% with isolated moderate-to-severe TR, and 22.9% with both conditions (*p* < 0.001; Figure 2, right). In univariable logistic regression, the unadjusted OR for isolated renal dysfunction compared with neither condition was 1.65 (95% CI 1.00–2.71; *p* = 0.050). Isolated moderate-to-severe TR was associated with higher unadjusted odds of one-year mortality (OR 1.77, 95% CI 1.15–2.73; *p* = 0.010), while the coexistence of both conditions showed the strongest association (OR 4.13, 95% CI 2.48–6.88; *p* < 0.001).

### 3.4. Multivariable, Interaction and Sensitivity Analyses

In the complete-case cohort of 1704 patients, the TR–renal phenotype remained associated with one-year all-cause mortality after multivariable adjustment (overall *p* = 0.005). Compared with patients with neither condition, isolated renal dysfunction (adjusted OR 1.26, 95% CI 0.74–2.15; *p* = 0.399) and isolated moderate-to-severe TR (adjusted OR 1.34, 95% CI 0.82–2.18; *p* = 0.239) were not significantly associated with mortality. In contrast, the coexistence of both conditions was associated with almost threefold higher adjusted odds of one-year mortality (adjusted OR 2.91, 95% CI 1.62–5.23; *p* < 0.001; Figure 2, left). In direct adjusted comparisons, the combined phenotype was also associated with higher odds of one-year mortality than isolated moderate-to-severe TR (adjusted OR 2.17, 95% CI 1.16–4.07; *p* = 0.015) and isolated renal dysfunction (adjusted OR 2.32, 95% CI 1.16–4.61; *p* = 0.017). When TR and renal dysfunction were analysed as separate predictors across the entire cohort, both were associated with one-year mortality in univariable analyses (moderate-to-severe TR: OR 2.14, 95% CI 1.51–3.03; *p* < 0.001; renal dysfunction: OR 2.09, 95% CI 1.45–3.03; *p* < 0.001). In a multivariable model including both conditions simultaneously and adjusted for the same prespecified covariates as the primary model (*n* = 1704), moderate-to-severe TR (adjusted OR 1.59, 95% CI 1.06–2.39; *p* = 0.024) and renal dysfunction (adjusted OR 1.57, 95% CI 1.05–2.35; *p* = 0.029) each remained independently associated with one-year mortality.

Increasing age (adjusted OR per year 1.07, 95% CI 1.04–1.11; *p* < 0.001), AF (adjusted OR 1.68, 95% CI 1.16–2.43; *p* = 0.006), chronic obstructive pulmonary disease (adjusted OR 2.01, 95% CI 1.27–3.18; *p* = 0.003), and PAD (adjusted OR 1.90, 95% CI 1.11–3.26; *p* = 0.020) were also associated with higher mortality. NYHA functional class was associated with mortality overall (*p* < 0.001), with significantly higher odds among patients in NYHA class IV compared with class I (adjusted OR 4.12, 95% CI 1.19–14.24; *p* = 0.025). Female sex was associated with lower odds of mortality (adjusted OR 0.65, 95% CI 0.45–0.94; *p* = 0.023). The model was significant overall (likelihood-ratio χ^2^ = 98.41; *p* < 0.001), demonstrated good calibration (Hosmer–Lemeshow *p* = 0.881), and had a Nagelkerke R^2^ of 0.124.

In the interaction model, the interaction between moderate-to-severe TR and renal dysfunction was not statistically significant (OR 1.73, 95% CI 0.77–3.90; *p* = 0.187), providing no evidence of effect modification on the multiplicative scale. When eGFR was analysed as a continuous variable, each 10 mL/min/1.73 m^2^ decrease was associated with higher one-year mortality in univariable analysis (OR 1.34, 95% CI 1.19–1.51; *p* < 0.001) and after multivariable adjustment (adjusted OR 1.24, 95% CI 1.08–1.42; *p* = 0.002). In the same multivariable model, moderate-to-severe TR remained independently associated with one-year mortality (adjusted OR 1.55, 95% CI 1.03–2.33; *p* = 0.035). Results remained consistent when LVEF was entered using the original four-level classification instead of the dichotomised classification of ≤45% versus >45%. In this sensitivity analysis, the combined phenotype remained significantly associated with one-year mortality (adjusted OR 2.90, 95% CI 1.61–5.21; *p* < 0.001), whereas neither isolated phenotype was significantly associated with the outcome. Findings were also unchanged after excluding the 41 patients receiving chronic dialysis before TAVR. In this sensitivity analysis, the combined TR–renal dysfunction phenotype remained independently associated with one-year mortality compared with neither condition (adjusted OR 2.94, 95% CI 1.61–5.37; *p* < 0.001; adjusted complete-case cohort, *n* = 1677). The TR × renal dysfunction interaction remained non-significant (*p* = 0.187).

### 3.5. Secondary Outcomes

Secondary outcomes are summarized in Table 2. Postprocedural length of hospital stay, in-hospital mortality, 30-day all-cause mortality, AKI, and the requirement for continuous venovenous hemodiafiltration differed significantly across the four phenotypes (all *p* < 0.001). Patients with both conditions had the longest hospital stay and the highest observed rates of all secondary clinical events. Patients with isolated renal dysfunction also had higher observed rates of early mortality and renal complications, whereas outcomes among patients with isolated moderate-to-severe TR were closer to those observed in patients with neither condition. These analyses were unadjusted and exploratory.

## 4. Discussion

The present study demonstrates that the coexistence of moderate-to-severe TR and renal dysfunction identifies a clinically relevant high-risk group among patients undergoing TAVR. Although only 5.9% of patients had both conditions, this group exhibited more advanced functional limitation, AF, concomitant mitral regurgitation, left ventricular dysfunction, and clinical markers of congestion. One-year mortality reached 22.9% compared with 6.7% among patients with neither condition, and the combined phenotype remained associated with almost threefold higher adjusted odds of mortality. In contrast, neither isolated renal dysfunction nor isolated moderate-to-severe TR remained significantly associated with mortality after multivariable adjustment. Patients with both conditions also had the highest unadjusted rates of early mortality, AKI, renal replacement therapy, and prolonged hospitalisation.

The prognostic relevance of TR after TAVR has been demonstrated across clinical trials and large registries [13,14]. However, TR frequently coexists with AF, mitral regurgitation, pulmonary hypertension, and right ventricular dysfunction, making it difficult to distinguish its direct contribution from that of the underlying cardiac disease. Previous studies found that the association between TR and mortality was attenuated after accounting for right ventricular function and that TR aetiology and persistent right ventricular dysfunction further stratified prognosis [15,16]. This is consistent with our finding that isolated TR was associated with mortality in the unadjusted analysis but not after comprehensive adjustment. Moreover, TR may improve, persist, or progress after TAVR, with residual or progressive TR carrying an unfavourable prognosis [17,18,19]. Because serial echocardiographic data were unavailable, the prognostic implications of postprocedural changes in TR could not be assessed in the present study.

Renal dysfunction is likewise associated with adverse outcomes after TAVR. The eGFR threshold of <45 mL/min/1.73 m^2^ used in our analysis has previously identified patients with increased short- and longer-term mortality [20]. Nevertheless, kidney function remains stable or improves in many patients following TAVR, whereas postprocedural deterioration and AKI are associated with increased mortality and subsequent dialysis [21,22,23]. In our cohort, isolated renal dysfunction was not significantly associated with one-year mortality after adjustment but remained associated with higher unadjusted rates of early renal complications. Baseline renal dysfunction may therefore identify reduced physiological reserve without fully capturing the potentially reversible haemodynamic component of renal impairment.

The particularly unfavourable outcome of patients with both conditions is biologically plausible. Moderate-to-severe TR may increase right atrial, systemic venous, and renal venous pressures, thereby reducing the effective renal perfusion gradient and promoting renal dysfunction. Impaired renal function can, in turn, promote sodium and fluid retention, neurohormonal activation, and systemic congestion, further increasing right ventricular preload and potentially aggravating TR. This bidirectional interaction may establish a self-perpetuating cardiorenal congestion cycle. Importantly, relief of the left-sided valvular obstruction by TAVR may not fully reverse advanced right-sided haemodynamic abnormalities or renal dysfunction, particularly when structural right ventricular remodelling and chronic venous congestion are already present. The combined phenotype may therefore reflect more advanced cardiac damage, impaired end-organ reserve, and greater susceptibility to decompensation and postprocedural complications. Cardiac-damage staging studies have demonstrated progressively worse outcomes as aortic stenosis extends from left-sided involvement to pulmonary hypertension, TR, and right ventricular dysfunction, while regression of such damage after valve replacement occurs in only a minority of patients [24,25]. Our findings also complement those of Kim et al., who identified particularly poor outcomes among patients with low-flow, low-gradient aortic stenosis and coexisting chronic kidney disease and right-sided extravalvular damage [10]. The present study extends this concept to a broader TAVR population and specifically examines the combination of TR and renal dysfunction. However, detailed measures of right ventricular function and pulmonary haemodynamics were not consistently available in the present study. We therefore cannot determine whether the excess risk observed in patients with coexisting TR and renal dysfunction reflects the combined clinical burden of these conditions or, at least in part, more advanced right ventricular failure and systemic venous congestion. Accordingly, our findings primarily support the prognostic value of jointly assessing two routinely available variables rather than establishing a distinct pathophysiological entity.

The absence of a significant multiplicative interaction is important. The categorical phenotype model demonstrated that patients with both conditions had substantially higher mortality than the reference group, whereas the interaction model examined whether their joint association exceeded that expected from their individual associations on the multiplicative scale. Consequently, the combined phenotype should be interpreted as a clinically relevant concentration of risk rather than evidence of statistical or biological synergy. The limited number of deaths in the combined group may also have reduced the precision of the interaction estimate.

Both TR severity and eGFR are routinely available before TAVR and may be considered jointly during preprocedural assessment. Identification of the combined phenotype should not be interpreted as a reason to withhold TAVR. Instead, it may prompt more detailed evaluation of right ventricular function, pulmonary pressures, volume status, and potentially reversible congestion, together with careful renal management and closer postprocedural surveillance. Serial reassessment of TR and renal function may be particularly relevant because their trajectories after TAVR provide prognostic information beyond a single baseline measurement. Whether earlier intervention or targeted treatment of persistent congestion and TR can improve outcomes requires prospective investigation.

## 5. Limitations

This study has several limitations. Its retrospective observational design precludes causal inference and leaves the possibility of residual and unmeasured confounding. One-year mortality status was unavailable for 481 of 2227 patients (21.6%); therefore, the primary outcome analysis was restricted to patients with documented follow-up. Baseline characteristics differed between patients with and without available one-year vital status, indicating that incomplete follow-up was not random and may have introduced selection bias. Accordingly, the observed mortality estimates should be interpreted with this limitation in mind. Furthermore, dates of death and reliable dates of last known contact were not available, preventing calculation of individual follow-up duration and precluding censoring-based time-to-event analyses. TR severity was based on routine echocardiographic assessment without central core-laboratory adjudication, and quantitative parameters of TR severity as well as detailed measures of right ventricular function and pulmonary haemodynamics were not consistently available. TR etiology was not systematically adjudicated, and primary and secondary TR could therefore not be reliably distinguished. TR severity was classified according to the contemporary three-grade echocardiographic classification used during the study period. Consequently, differentiation between severe, massive, and torrential TR according to the more recent guideline classification was not possible.

Renal dysfunction was defined using a single preprocedural eGFR measurement, and the underlying etiology of renal dysfunction was not systematically available and could therefore not be evaluated. Longitudinal changes in renal function and TR after TAVR could not be assessed. In addition, AKI was ascertained retrospectively from clinically obtained serum creatinine measurements rather than according to a standardized postprocedural surveillance protocol. Differences in the timing or frequency of creatinine measurements across phenotypes therefore cannot be excluded and may have influenced AKI detection. Procedural contrast volume was not systematically captured and could therefore not be accounted for when evaluating post-TAVR AKI. The relatively small number of patients and deaths in the combined phenotype may also have limited the precision and statistical power of the interaction analysis. Furthermore, the STS score was unavailable in approximately 45% of patients, likely reflecting temporal changes in routine calculation and documentation; it was therefore not included in the multivariable model to avoid a substantial reduction in sample size, and comparisons involving the STS score should be interpreted cautiously. Baseline medication data were not available, precluding assessment of their potential association with outcomes. Finally, missing data were handled using complete-case analysis, secondary outcomes were analysed on an unadjusted and exploratory basis, and cause-specific mortality was unavailable, precluding differentiation between cardiovascular deaths, deaths related to renal failure, and other causes.

## 6. Conclusions

Coexisting moderate-to-severe TR and renal dysfunction identify a clinically relevant high-risk group after TAVR, with higher adjusted one-year mortality than either condition alone. Combined assessment of TR and renal function may therefore provide useful incremental information for preprocedural risk stratification. These findings should be interpreted as prognostic rather than as evidence of a distinct biological TR–renal entity.

## Figures and Tables

**Figure 1:**
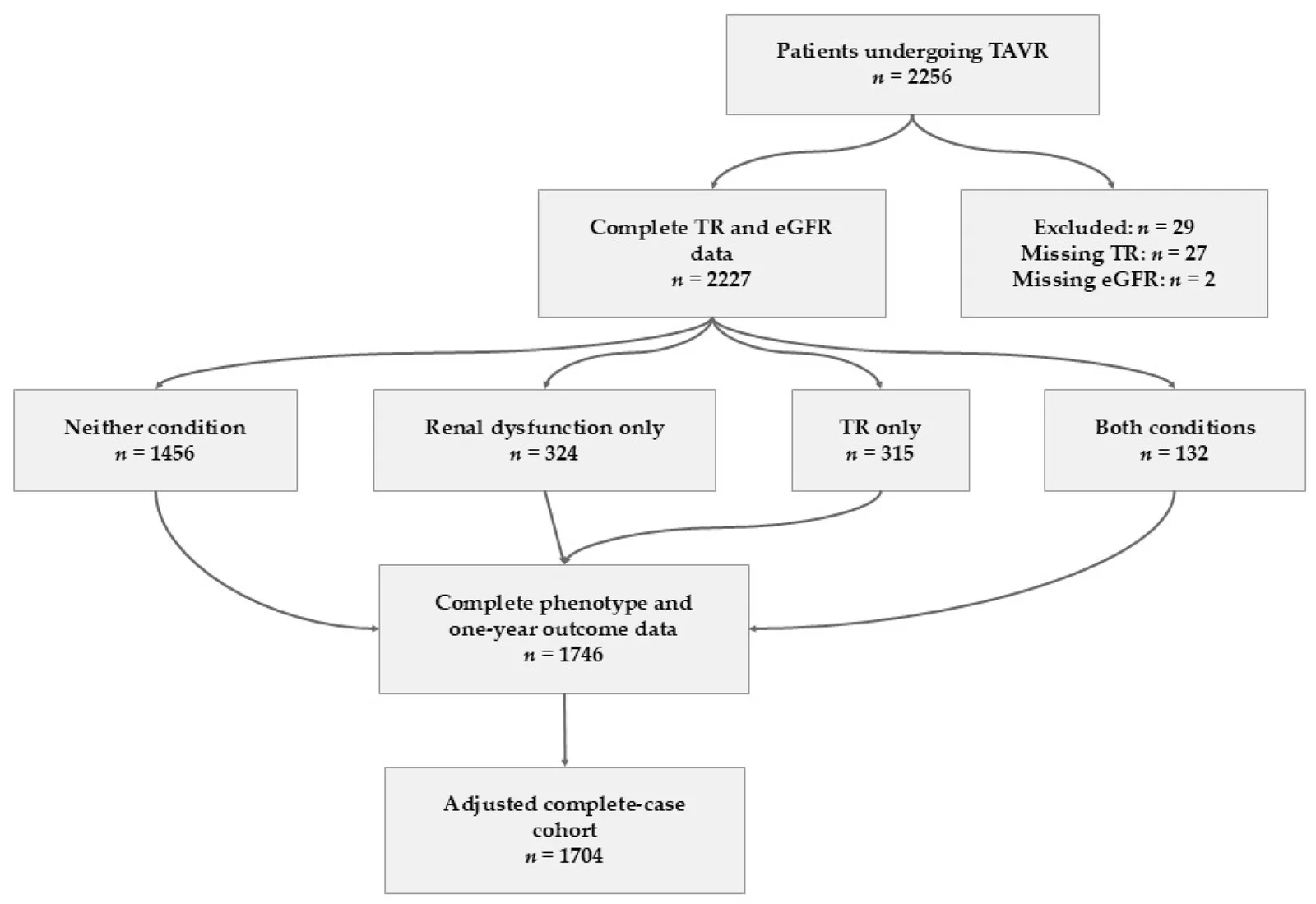
Flowchart of patient selection and allocation to the four TR–renal phenotypes. eGFR, estimated glomerular filtration rate; TAVR, transcatheter aortic valve replacement; TR, tricuspid regurgitation.

**Figure 2:**
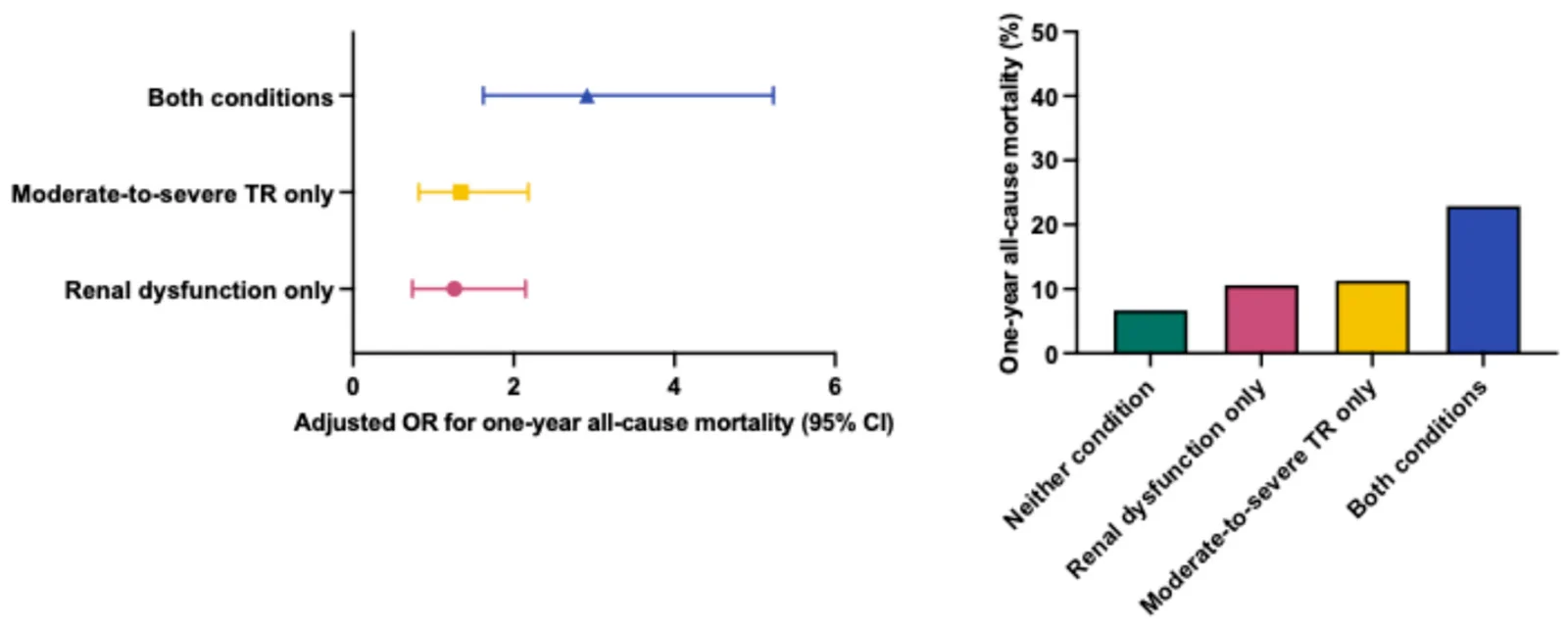
One-year all-cause mortality according to TR–renal phenotype. (**Left**) Adjusted odds ratios with 95% confidence intervals for one-year all-cause mortality, with patients with neither condition serving as the reference group. (**Right**) Observed one-year all-cause mortality rates across the four TR–renal phenotypes (overall *p* < 0.001). OR, odds ratio; CI, confidence interval; TR, tricuspid regurgitation.

**Table 1:** Baseline characteristics according to TR–renal phenotype. Values are presented as mean ± standard deviation, median [interquartile range], or *n* (%), as appropriate. Continuous variables were compared using the Kruskal–Wallis test and categorical variables using Pearson’s χ^2^ test. When the overall Kruskal–Wallis test was significant, pairwise comparisons were performed with Bonferroni adjustment. NT-proBNP, N-terminal pro-B-type natriuretic peptide; NYHA, New York Heart Association; STS, Society of Thoracic Surgeons; TR, tricuspid regurgitation.

Characteristic	*n*	Neither Condition (*n* = 1456)	Renal Dysfunction only (*n* = 324)	TR Only (*n* = 315)	Both Conditions (*n* = 132)	*p*-Value
**Demographics and functional status**						
Age, years	2227	79.0 ± 6.4	81.3 ± 6.4	80.7 ± 6.0	80.6 ± 6.4	<0.001
Male sex	2227	821 (56.4)	142 (43.8)	166 (52.7)	63 (47.7)	<0.001
Body mass index, kg/m^2^	2215	28.5 ± 5.2	29.4 ± 5.8	27.5 ± 5.0	29.1 ± 5.4	<0.001
NYHA functional class III/IV, *n* (%)	2161	985 (70.7)	257 (84.0)	227 (74.2)	112 (88.2)	<0.001
**Comorbidities**						
Arterial hypertension	2226	1298 (89.2)	299 (92.3)	268 (85.1)	115 (87.1)	0.029
Hyperlipidaemia	2225	1011 (69.4)	226 (70.0)	194 (61.8)	90 (68.2)	0.059
Smoking history	2227	240 (16.5)	45 (13.9)	42 (13.3)	21 (15.9)	0.421
Diabetes mellitus	2226	556 (38.2)	160 (49.5)	108 (34.3)	69 (52.3)	<0.001
Insulin-dependent diabetes mellitus	2227	221 (15.2)	85 (26.2)	37 (11.7)	43 (32.6)	<0.001
Coronary artery disease	2227	797 (54.7)	209 (64.5)	167 (53.0)	89 (67.4)	<0.001
Prior myocardial infarction	2226	155 (10.7)	53 (16.4)	35 (11.1)	24 (18.2)	0.004
Prior coronary artery bypass grafting	2226	81 (5.6)	23 (7.1)	16 (5.1)	14 (10.6)	0.086
Peripheral artery disease	2226	185 (12.7)	65 (20.1)	28 (8.9)	18 (13.6)	<0.001
Prior stroke	2224	67 (4.6)	17 (5.3)	8 (2.5)	7 (5.3)	0.319
Chronic obstructive pulmonary disease	2226	169 (11.6)	35 (10.8)	27 (8.6)	18 (13.6)	0.350
Malignancy	2226	155 (10.7)	37 (11.4)	25 (7.9)	10 (7.6)	0.307
Atrial fibrillation	2227	389 (26.7)	146 (45.1)	191 (60.6)	90 (68.2)	<0.001
Pre-existing permanent pacemaker	2223	103 (7.1)	49 (15.2)	53 (16.8)	27 (20.5)	<0.001
**Congestion and laboratory findings**						
Pleural effusion	2227	104 (7.1)	35 (10.8)	47 (14.9)	44 (33.3)	<0.001
Radiographic pulmonary congestion	2227	66 (4.5)	23 (7.1)	27 (8.6)	18 (13.6)	<0.001
Creatinine, mg/dL	2226	0.9 [0.7–1.0]	1.4 [1.2–1.8]	0.9 [0.7–1.0]	1.6 [1.3–2.2]	<0.001
C-reactive protein, mg/dL	2221	2.7 [1.1–6.4]	5.1 [1.9–12.8]	3.2 [1.4–8.9]	6.9 [2.6–18.6]	<0.001
Haemoglobin, g/L	2227	128.3 ± 17.3	116.7 ± 17.4	124.8 ± 17.6	116.5 ± 17.2	<0.001
Sodium, mmol/L	2208	140.3 ± 3.5	140.1 ± 4.2	140.3 ± 4.3	140.2 ± 4.1	0.875
NT-proBNP, pg/mL	1702	928.0 [390.0–2432.0]	2199.0 [648.0–6426.0]	2190.0 [950.0–4320.0]	5559.0 [2396.0–19,895.0]	<0.001
**Risk scores and echocardiography**						
EuroSCORE II, %	1974	3.7 [2.4–6.5]	7.7 [5.0–13.1]	5.0 [3.0–9.2]	10.8 [6.5–20.8]	<0.001
STS score, %	1214	3.6 [2.1–9.0]	6.2 [3.5–11.9]	4.8 [2.7–11.2]	8.9 [4.4–14.9]	<0.001
Left ventricular ejection fraction ≤ 45%	2201	292 (20.3)	91 (28.5)	84 (26.7)	55 (41.7)	<0.001
Peak aortic valve velocity, m/s	1673	4.2 ± 0.7	4.0 ± 0.7	4.1 ± 0.7	3.9 ± 0.9	<0.001
Mean aortic valve gradient, mmHg	2038	50.8 ± 19.7	47.7 ± 19.9	45.1 ± 16.6	44.6 ± 20.6	<0.001
Aortic valve area, cm^2^	2095	0.8 [0.6–0.9]	0.8 [0.6–0.9]	0.7 [0.6–0.8]	0.7 [0.6–0.8]	<0.001
At least moderate mitral regurgitation	2225	308 (21.2)	99 (30.6)	169 (53.7)	93 (70.5)	<0.001

**Table 2:** Clinical outcomes according to TR–renal phenotype. Values are presented as median [interquartile range] or *n* (%), as appropriate. Continuous variables were compared using the Kruskal–Wallis test and categorical variables using Pearson’s χ^2^ test. For one-year mortality, denominators represent patients with available one-year vital status within each phenotype. TR, tricuspid regurgitation.

Characteristic	*n*	Neither Condition (*n* = 1456)	Renal Dysfunction Only (*n* = 324)	TR Only (*n* = 315)	Both Conditions (*n* = 132)	*p*-Value
**Clinical outcomes**						
Postprocedural hospital stay, days	2166	7.0 [5.0–10.0]	8.0 [6.0–12.0]	7.0 [5.0–11.0]	10.0 [7.0–13.0]	<0.001
In-hospital mortality	2227	19 (1.3)	19 (5.9)	8 (2.5)	10 (7.6)	<0.001
30-day all-cause mortality	2227	27 (1.9)	20 (6.2)	9 (2.9)	12 (9.1)	<0.001
Acute kidney injury	2227	22 (1.5)	46 (14.2)	4 (1.3)	25 (18.9)	<0.001
Continuous venovenous haemodiafiltration	2227	9 (0.6)	26 (8.0)	1 (0.3)	14 (10.6)	<0.001
One-year all-cause mortality, *n*/*N* (%)	1746	77/1149 (6.7)	22/208 (10.6)	32/284 (11.3)	24/105 (22.9)	<0.001

## Data Availability

The data that support the findings of this study are available from the corresponding author upon reasonable request, subject to applicable ethical and data-protection restrictions.

## Supplementary Materials

The following supporting information can be downloaded at: https://www.mdpi.com/article/10.3390/jcdd13100520/s1, Table S1. Baseline characteristics according to availability of one-year vital status.
