# Supplementary material for: Coexisting Moderate-to-Severe Tricuspid Regurgitation and Renal Dysfunction Identify a High-Risk Group After Transcatheter Aortic Valve Replacement
Source: J Cardiovasc Dev Dis. 2026 Oct 16;13(10):520. doi: 10.3390/jcdd13100520 (PMC13705320; doi:10.3390/jcdd13100520)
Supplement: Supplementary file 1 [file jcdd-13-00520-s001.zip › jcdd-4577706-supplementary.pdf]

Supplementary material

Supplementary Table S1. Baseline characteristics according to availability of one-year vital status. Values are presented as mean ± standard deviation, median [interquartile range], or *n* (%), as appropriate. Percentages are calculated among patients with available data for the respective variable. Continuous variables were compared using the Mann–Whitney U test and categorical variables using Pearson's χ^2 test. The p-value for TR–renal phenotype represents the overall comparison of phenotype distribution between patients with and without available one-year vital status. eGFR, estimated glomerular filtration rate; LVEF, left ventricular ejection fraction; NT-proBNP, N-terminal pro-B-type natriuretic peptide; NYHA, New York Heart Association; STS, Society of Thoracic Surgeons; TR, tricuspid regurgitation.

Characteristic	*n*	One-year vital status available (*n*=1,746)	One-year vital status unavailable (*n*=481)	p-value
Demographics and functional status				
Age, years	2,227	79.3 ± 6.4	81.1 ± 6.3	<0.001
Male sex, *n* (%)	2,227	933 (53.4)	259 (53.8)	0.873
Body mass index, kg/m²	2,215	28.7 ± 5.4	27.6 ± 4.9	<0.001
NYHA functional class III/IV, *n* (%)	2,161	1,190 (69.3)	391 (94.2)	<0.001
Comorbidities				
Arterial hypertension, *n* (%)	2,226	1,523 (87.3)	457 (95.0)	<0.001
Hyperlipidaemia, *n* (%)	2,225	1,218 (69.8)	303 (63.0)	0.004
Smoking history, *n* (%)	2,227	267 (15.3)	81 (16.8)	0.408
Diabetes mellitus, *n* (%)	2,226	733 (42.0)	160 (33.3)	<0.001
Insulin-dependent diabetes mellitus, *n* (%)	2,227	308 (17.6)	78 (16.2)	0.465
Coronary artery disease, *n* (%)	2,227	957 (54.8)	305 (63.4)	<0.001
Prior myocardial infarction, *n* (%)	2,226	196 (11.2)	71 (14.8)	0.035
Prior coronary artery bypass grafting, *n* (%)	2,226	105 (6.0)	29 (6.0)	0.992
Peripheral artery disease, *n* (%)	2,226	130 (7.4)	166 (34.5)	<0.001
Prior stroke, *n* (%)	2,224	56 (3.2)	43 (9.0)	<0.001
Chronic obstructive pulmonary disease, *n* (%)	2,226	183 (10.5)	66 (13.8)	0.044
Malignancy, *n* (%)	2,226	108 (6.2)	119 (24.7)	<0.001
Atrial fibrillation, *n* (%)	2,227	618 (35.4)	198 (41.2)	0.020
Pre-existing permanent pacemaker, *n* (%)	2,223	187 (10.7)	45 (9.4)	0.390
Congestion and laboratory findings				
Pleural effusion, *n* (%)	2,227	201 (11.5)	29 (6.0)	<0.001
Radiographic pulmonary congestion, *n* (%)	2,227	97 (5.6)	37 (7.7)	0.081
Creatinine, mg/dL	2,226	0.90 [0.73–1.13]	1.08 [0.86–1.41]	<0.001
C-reactive protein, mg/dL	2,221	3.1 [1.2–7.9]	3.8 [1.6–10.6]	0.002
Haemoglobin, g/L	2,227	125.9 ± 17.8	123.7 ± 18.4	0.005
Sodium, mmol/L	2,208	140.8 ± 3.6	138.2 ± 3.8	<0.001
NT-proBNP, pg/mL	1,702	1,627.5 [602.8–3,685.5]	653.0 [248.0–2,181.2]	<0.001
Risk scores and echocardiography				

**Characteristic**	***n***	**One-year vital status available (*n*=1,746)**	**One-year vital status unavailable (*n*=481)**	**p-value**
EuroSCORE II, %	1,974	4.75 [2.76–9.04]	4.00 [2.80–6.33]	<0.001
STS score, %	1,214	5.12 [2.30–11.00]	3.30 [2.50–5.30]	<0.001
LVEF ≤45%, *n* (%)	2,201	448 (25.8)	74 (15.9)	<0.001
Peak aortic valve velocity, m/s	1,673	4.16 ± 0.69	3.88 ± 0.82	<0.001
Mean aortic valve gradient, mmHg	2,038	45.1 ± 15.6	64.8 ± 24.4	<0.001
Aortic valve area, cm^2	2,095	0.70 [0.60–0.90]	0.80 [0.70–0.90]	<0.001
At least moderate mitral regurgitation, *n* (%)	2,225	572 (32.8)	97 (20.2)	<0.001
Renal function and TR				
eGFR, mL/min/1.73 m^2	2,227	60.0 [51.3–60.0]	57.0 [41.0–60.0]	<0.001
eGFR <45 mL/min/1.73 m^2, *n* (%)	2,227	313 (17.9)	143 (29.7)	<0.001
Moderate-to-severe TR, *n* (%)	2,227	389 (22.3)	58 (12.1)	<0.001
TR–renal phenotype				<0.001
Neither condition, *n* (%)	2,227	1,149 (65.8)	307 (63.8)	
Isolated renal dysfunction, *n* (%)	2,227	208 (11.9)	116 (24.1)	
Isolated moderate-to-severe TR, *n* (%)	2,227	284 (16.3)	31 (6.4)	
Both conditions, *n* (%)	2,227	105 (6.0)	27 (5.6)	
